# Thermodynamically enabled and reaction attuned estimation of metabolic fluxes

**DOI:** 10.1016/j.isci.2026.115822

**Published:** 2026-04-21

**Authors:** Nicolás Améstica-Toledo, Maximiliano Farías-Miño, Raúl Conejeros, David Tourigny, Marcelo Rivas-Astroza

**Affiliations:** 1 Universidad Tecnológica Metropolitana, Departamento de Biotecnología, Santiago, Chile; 2 Pontificia Universidad Católica de Valparaíso, Escuela de Ingeniería Bioquímica, Valparaíso, Chile; 3 School of Mathematics, University of Birmingham, Birmingham, UK

**Keywords:** biochemistry, human metabolism, flux data

## Abstract

Mathematical models of metabolism based on mass-balance constraints are essential for analyzing cellular biochemistry, but their predictions are often compromised by thermodynamically infeasible flux cycles—unrealistic loops of biochemical reactions that circulate continuously without external energy input. Here, we introduce Teraflux, a method that generates transcriptome-specific metabolic flux estimations free of these cycles. Teraflux maximizes Shannon entropy weighted by gene expression data while enforcing relaxed thermodynamic consistency. By distinguishing between chemical potentials and reaction rates, it uses flux constraints to encode biological irreversibility without overfitting the chemical potential space. Across diverse experimental conditions in *Escherichia coli*, Teraflux achieves high prediction accuracy, preserves biologically essential feasible cycles like the glyoxylate shunt, and prevents implausible metabolic states such as artificial glucose excretion. Furthermore, thermodynamic variability analysis confirms that Teraflux’s predictions fundamentally align with physiological limits on intracellular metabolite concentrations. Teraflux provides a robust, computationally efficient tool for obtaining reliable, condition-specific fluxomes.

## Highlights

• Teraflux estimates transcriptome-specific fluxomes without infeasible cycles
• Maximizes entropy while enforcing a relaxed thermodynamic consistency
• Accurately predicts fluxes and preserves feasible loops like the glyoxylate shunt
• Generates robust, condition-specific fluxes on genome-scale models in seconds

## Graphical abstract

**Figure:**
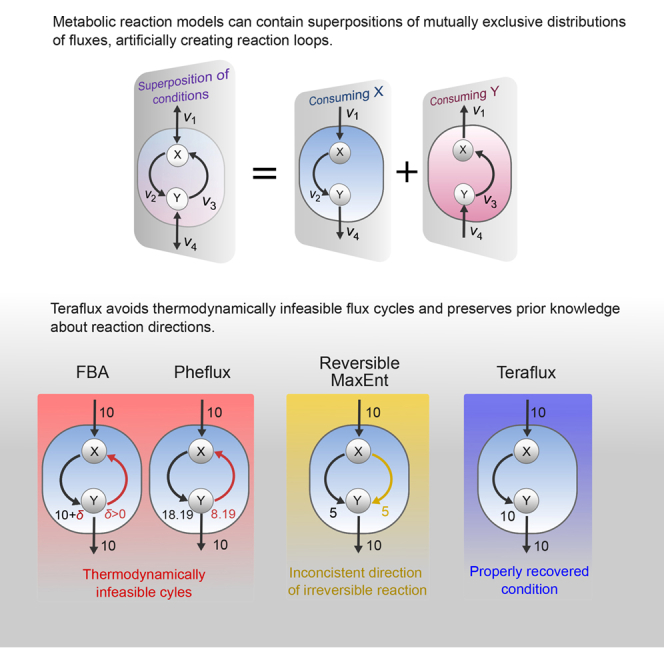

## Introduction

Mathematical models of metabolism have become indispensable for understanding the intricate network of biochemical reactions in biological systems.^1^ Among these, constraint-based models are prominent for their ability to predict metabolic phenotypes—such as growth rates, metabolic fluxes, and gene essentiality—using stoichiometric and physiological constraints.^1^^,^^2^ By representing the entirety of an organism’s known biochemical reactions, these models enable the simulation and analysis of complex cellular behaviors.^2^

A critical challenge in constraint-based modeling, however, is the possibility of thermodynamically infeasible cycles within the metabolic network.^3^ These are closed loops of reactions that violate fundamental thermodynamic principles.^4^^,^^5^ They arise because mass balance constraints alone are insufficient to prevent unrealistic scenarios where arbitrarily large fluxes circulate between metabolites, effectively generating energy without any external input.^6^ Such thermodynamically inconsistent behavior can lead to inaccurate estimations of metabolic fluxes, a distorted representation of cellular behavior, and misinterpretations of experimental data.^7^^,^^8^^,^^9^ These thermodynamic artifacts stem from incomplete or inaccurate knowledge of the Gibbs free energy changes associated with reactions under *in vivo* conditions,^10^ causing models to inadvertently incorporate reaction cycles that defy the principles of energy conservation and entropy production.^11^ The consequences of these infeasible cycles extend beyond theoretical inconsistencies; they can lead to inaccurate predictions of gene essentiality and misguided metabolic engineering strategies.^12^^,^^13^^,^^14^

Various approaches have been developed to address thermodynamically infeasible cycles. These range from manually curating reaction thermodynamics based on experimental data^15^^,^^16^^,^^17^ to integrating complex non-linear thermodynamic constraints directly into the model formulation^18^ or developing algorithms to identify and eliminate the cycles.^7^^,^^19^^,^^20^^,^^21^^,^^22^ While successful to an extent, these methods often suffer from limitations, including reliance on incomplete thermodynamic data,^23^ high computational complexity (for instance, some formulations framed as MILP problems have been shown to be NP-hard^24^), or the potential for over-constraining the model by removing biologically important, thermodynamically feasible cycles.^25^

Alternatively, Fleming et al.^26^ developed a constraint-based model that ensures thermodynamically consistent flux distributions by solving a convex optimization problem based on a variational principle, thereby avoiding complex non-linear constraints. Application of this approach includes the identification of therapeutic targets to mitigate metabolic dysfunction,^27^ and the characterization of human dopaminergic neuronal metabolism.^28^ A key feature of this framework is its ability to integrate weighting parameters to each flux, opening the possibility of refining flux estimations. The model treats all non-exchange reactions as reversible, which aligns with the chemical principle that all reactions are theoretically reversible due to their finite equilibrium constants.^29^ However, this assumption conflicts with biological reality, where many reactions are practically irreversible. Key steps in pathways like glycolysis, for example, are effectively unidirectional due to a combination of large negative free energy changes (Δ*G*) and high activation energy barriers that necessitate enzymatic catalysis.^30^^,^^31^^,^^32^ While this directionality can be enforced on the model by setting lower flux bounds to zero, this solution compromises the method’s strict thermodynamic consistency. It leads to a condition similar to that which other authors have termed relaxed thermodynamic consistency, where a reaction can have zero flux even when the thermodynamic driving force for spontaneous reactivity exists.^6^^,^^24^^,^^33^

More broadly, the formulation proposed by Fleming et al.^26^ falls within a type of constraint-based models that use the principle of maximum entropy to infer fluxomes.^34^ This principle has been applied to infer individual reaction fluxes,^25^ the distribution of metabolically heterogeneous cellular populations,^35^^,^^36^ the distribution of elementary flux modes,^37^^,^^38^ and regulatory strategies maximizing growth.^39^ Within this context, and building on the goal of integrating omics data, Pheflux, a method from Gonzalez et al.,^40^ estimates phenotype-specific fluxes using transcriptomics. Pheflux operates by maximizing Shannon’s entropy based on a probability distribution where a reaction’s flux is proportional to the expression of its associated genes. This process yields a unique flux distribution that is maximally consistent with the transcriptome. The mathematical objective of Pheflux is analogous to that used by Fleming et al.,^26^ highlighting the conceptual link between the two approaches. However, they differ significantly in how they handle reaction directionality. While the method of Fleming et al. was designed specifically to achieve global thermodynamic consistency, Pheflux relies on prior knowledge to selectively define which reactions are irreversible. Consequently, the original Pheflux formulation does not inherently prevent all thermodynamically impossible flux cycles, which was the central challenge Fleming et al. method sought to address.

In this work, we introduce Teraflux (thermodynamically enabled and reaction attuned FLUX-ome estimations), a framework designed to resolve the trade-offs between these preceding methods. Teraflux effectively unifies their core strengths into a single, cohesive model. From the approach of Fleming et al., it adopts the foundational structure of splitting all reactions into forward and reverse components, creating a thermodynamically aware framework. Inspired by Pheflux, it then uses gene expression levels to directly inform the parameters of the objective function, ensuring the resulting flux predictions are phenotype-specific. Crucially, Teraflux integrates a final, pragmatic layer of biological realism by enforcing hard constraints on reactions known to be irreversible. We demonstrate that this hybrid design, despite formally meeting the criteria for relaxed thermodynamic consistency,^6^^,^^24^ is sufficient to prevent the formation of thermodynamically infeasible cycles. Teraflux thus provides phenotype-specific flux estimations without sacrificing essential thermodynamic integrity.

## Results

### Model description and comparison to alternative methods

**Figure 1:**
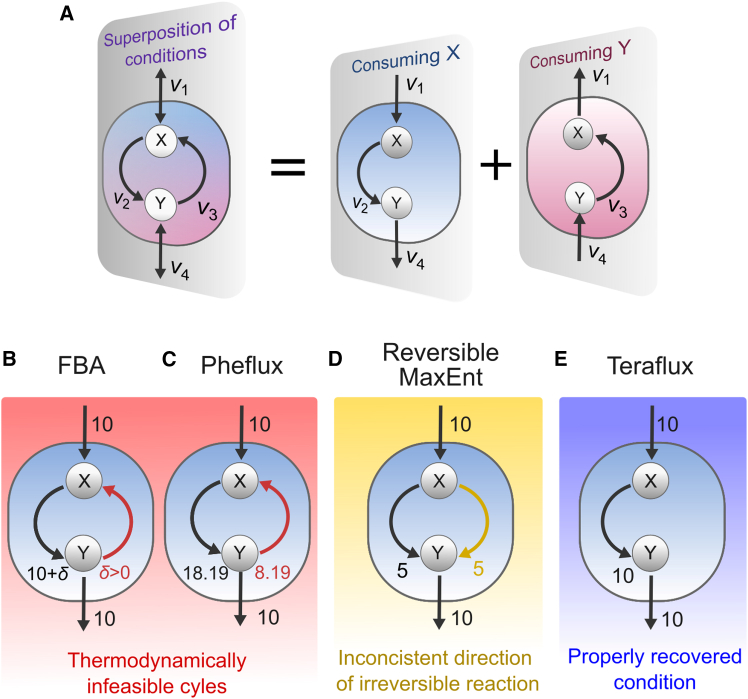
Accurate prediction of metabolic fluxes using Teraflux (A) A general network with a loop between X and Y. Modeling X consumption and fixing *v*_1_ = 10 (mmol g^−1^ h^−1^) reveals limitations in various methods. (B) FBA predicts a thermodynamically infeasible cycle by transforming Y back into X via *v*_3_. FBA is particularly sensitive to infeasible cycles as the amount of recirculated flux, *δ*, can be any positive value. (C) Although less pronounced, Pheflux still predicts recirculation fluxes between X and Y. (D) The method of Fleming et al. (reversible MaxEnt) changes the direction of the irreversible reaction *v*_3_. (E) Only Teraflux properly recovers the condition where X is converted into Y exclusively via reaction 2.

To illustrate the advantages of Teraflux over existing methods, we constructed a simplified metabolic network (Figure 1A) featuring a reversible loop between metabolites X and Y. Although simple, this network can exhibit thermodynamically infeasible flux distributions, as mass balance constraints alone are insufficient to prevent unrealistic flux cycles between X and Y. We analyzed the difference between methods by modeling X consumption and fixing the uptake flux *v*_1_ = 10 (mmol g^−1^ h^−1^). Flux balance analysis (FBA), when maximizing the output flux *v*_4_, yields a wide range of possible flux distributions where an infeasible cycle can carry arbitrarily large flux (Figure 1B). Maximizing flux entropy conditioned on a uniform gene expression pattern (Pheflux^40^), while respecting defined reaction directions, still predicts the energetically wasteful recirculation of flux (Figure 1C). The method by Fleming et al.,^26^ which requires all internal reactions to be reversible, avoids the recirculation but does so by reversing the direction of the irreversible reaction *v*_3_, contradicting prior knowledge (Figure 1D). In contrast, Teraflux, using the same uniform gene expression data, predicts a unique flux distribution with zero flux through the problematic reaction *v*_3_, aligning with known reaction directionality and avoiding the infeasible cycle (Figure 1E).

### Sampling fluxomes free of thermodynamically infeasible cycles

Extremely large fluxes are a telltale sign of thermodynamically inconsistent fluxomes. A clear example of this occurs in the Krebs cycle of the *E. coli* core model. Here, an infeasible cycle can form between two reactions: FRD7, which converts fumarate to succinate, and SUCDi, which catalyzes the reverse reaction. For the network to be thermodynamically feasible, at least one of these reactions must carry zero net flux.

**Figure 2:**
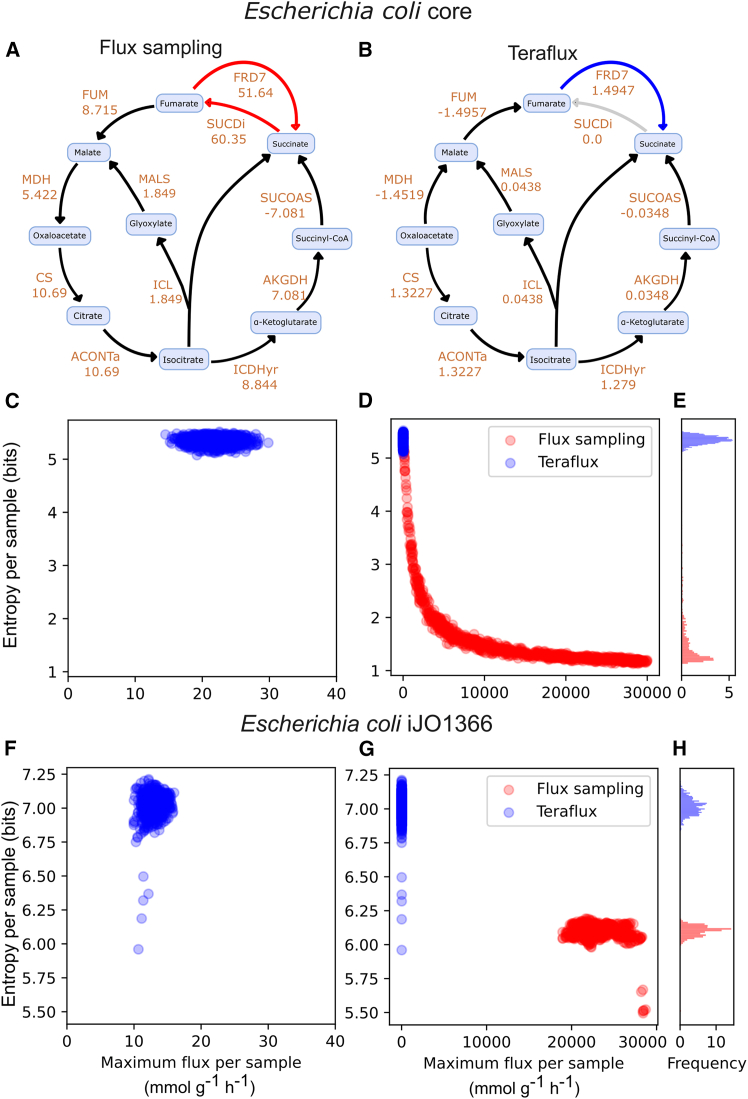
Sampling of fluxomes using Teraflux Side-by-side comparisons of Teraflux (blue) and Flux Sampling (red) for *E. coli* core (A–E) and iJO1366 (F–H). (A) and (B) illustrative flux maps for the *E. coli* core model highlighting how FBA and Teraflux diverge. Only Teraflux enforces mutual exclusivity between FRD7 and SUCDi; activating both would create a thermodynamically infeasible cycle. To improve visibility, not all co-substrates or alternative active reactions (e.g., the transport reaction AKGt2rpp that consumes alpha-ketogluratate) are shown. (C) and (F) show sample entropy vs. maximum flux in a zoomed-in scale, revealing Teraflux’s higher-entropy solutions that avoid extreme flux values. (D) and (G) expand the flux axis, highlighting how flux sampling can reach the 30,000 mmol g^−1^ h^−1^ boundary, which is indicative of thermodynamically infeasible cycles. (E) and (H) present histograms of sample entropies, underscoring Teraflux’s tendency toward higher-entropy (more diverse) flux states compared to flux sampling.

However, when we performed flux sampling with wide bounds (±30,000 mmol g^−1^ h^−1^) and limited glucose uptake (≤10 mmol g^−1^ h^−1^), the resulting fluxome incorrectly assigned simultaneous, non-zero fluxes to both FRD7 and SUCDi (Figure 2A). We then tested if Teraflux could correct this. Using the generated flux magnitudes as a proxy for gene expression (*g* = |*v*|), Teraflux predicted zero flux for SUCDi, successfully preventing the thermodynamically inconsistent cycle (Figure 2B). This demonstrates how Teraflux avoids the large, thermodynamically infeasible cycles that can appear in standard sampling, particularly when reaction bounds are non-restrictive.

To systematically compare the outputs of standard flux sampling and Teraflux, we designed a simulation pipeline using two genome-scale *E. coli* models (core and iJO1366). First, we generated 1,000 flux vectors (*v*) for each model using uniform flux sampling with wide bounds (±30,000 mmol g^−1^ h^−1^) and limited glucose uptake (≤10 mmol g^−1^ h^−1^). Next, each flux vector was used to simulate a corresponding gene expression profile by setting the expression (*g*) equal to the flux magnitude (*g* = |*v*|). Finally, these simulated profiles and their respective glucose uptake rates were used as inputs for Teraflux.

Our analysis of the maximum flux magnitudes revealed a stark difference. Flux sampling frequently produced fluxomes with values reaching the upper bound of 30,000 mmol g^−1^ h^−1^ (Figures 2D and 2G). In contrast, Teraflux never produced fluxes close to these bounds in either the core (Figure 2C) or iJO1366 model (Figure 2F). Consequently, this affected the fluxome entropy. The large flux values from sampling were concentrated in a few thermodynamically infeasible cycles, resulting in significantly lower average entropy compared to Teraflux for the *E. coli* core model (1.6 bits vs. 5.37 bits; *p* value $<1\times 10^{-8}$, Welch’s *t* test; Figure 2E). This pattern was replicated in the larger iJO1366 model, where Teraflux again yielded a higher average entropy (7.61 bits vs. 6.20 bits; *p* value$<1\times 10^{-8}$, Welch’s *t* test; Figure 2H).

**Figure 3:**
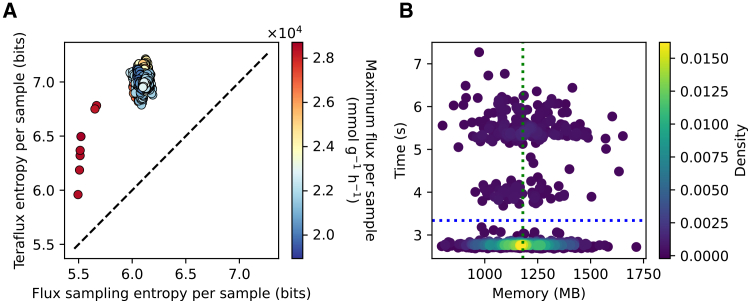
Teraflux generates higher-entropy fluxomes compared to flux sampling within seconds using 1.1 GB of RAM (A) Entropy of fluxomes computed using Teraflux and standard flux sampling. The fluxome generated by flux sampling was used as gene expression input data for Teraflux. The dotted line represents the *y* = *x* relationship. (B) Computational resource requirements of Teraflux: memory usage (*x* axis) versus computation time (*y* axis) for generating fluxomes. The dotted blue and green lines denote average time and memory, respectively.

Although a few Teraflux fluxomes exhibit lower entropy than their corresponding flux sampling solutions (see Figure 2G), the overall trend is the opposite. As the scatterplot reveals (Figure 3A), every solution from flux sampling corresponds to a Teraflux counterpart with higher entropy. For any new method to be practical, however, its computational demands must be reasonable. To be useful in real-world scenarios, such as analyzing large datasets, Teraflux must provide inferences on standard hardware. We evaluated this by measuring the resources required to generate the fluxomes for the genome-scale *E. coli* iJO1366 model. The results show that Teraflux takes on average less than 3.5 s and requires less than 1.25 GB of RAM per simulation (see Figure 3B).

### Preservation of thermodynamically feasible cycles

**Figure 4:**
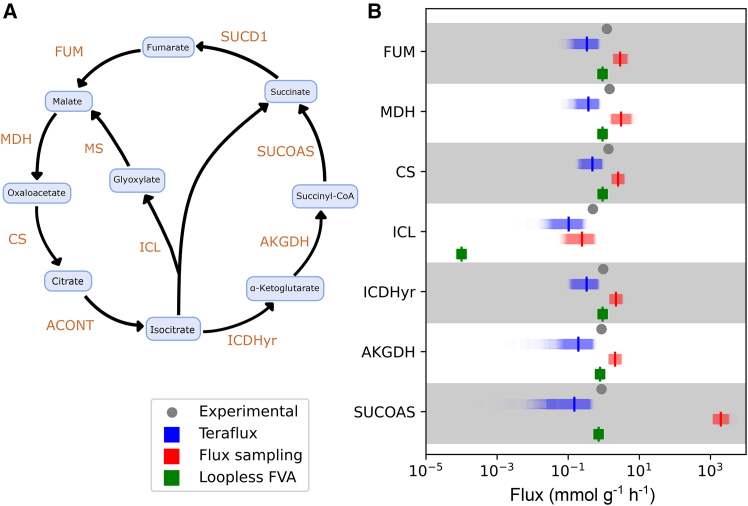
Fluxes through thermodynamically feasible and infeasible cycles in *E. coli* iJO1366 (A) Depiction of the Krebs and glyoxylate cycles. Whereas SUCOAS is a member of a set of reactions forming a thermodynamically infeasible cycle, ICL consumes energy to transform isocitrate back into glyoxylate and succinate, forming a thermodynamically feasible cycle. (B) Experimental ^13^C from Ishii et al.^41^ (culture condition 0.1 h^−1^) and predicted fluxes. Flux sampling, by being prone to infeasible cycle artifacts, overestimates SUCOAS flux by three orders of magnitude. Loopless FVA, based on a flux economy assumption, eliminates flux through all cycles, erroneously predicting zero flux via ICL. Only Teraflux consistently predicts the flux values.

A major drawback of some methods for removing infeasible cycles, like Loopless Flux Variability Analysis, is that they can eliminate all cycles, including those that are thermodynamically feasible and biologically essential. A key example is the glyoxylate shunt in *E. coli* (Figure 4A), a pathway comprised of isocitrate lyase (ICL) and malate synthase (MS) that is essential for carbon conservation.^42^ While the glyoxylate shunt is typically inactive when glucose is available, multiple studies have confirmed its activity and thermodynamic feasibility when *E. coli* is cultured at low growth rates.^41^^,^^43^^,^^44^ We investigated whether Teraflux could accurately predict its flux using ^13^C-derived *E. coli* flux data from Ishii et al.^41^ (growth rate: 0.1 h^−1^). Since Teraflux requires transcriptomic data, we used the simulated gene expression data of the previous section. We also computed fluxes with two tools widely available via the COBRApy library^45^^,^^46^: Flux sampling and loopless FVA, which is based on the minimization of the L-1 norm.

Our results (Figure 4B) show that Teraflux correctly predicts flux through ICL, unlike loopless FVA which predicts zero activity. While the average ICL flux from flux sampling is similar to Teraflux’s prediction, the methods diverge significantly for succinyl-CoA synthetase (SUCOAS). Teraflux’s SUCOAS prediction aligns with experimental data, whereas flux sampling overestimates it by three orders of magnitude because this reaction participates in a thermodynamically infeasible cycle. Overall, only Teraflux exhibits a consistent correlation (0.889 Pearson correlation; *p* value$<8\times 10^{-3}$) with the experimental data.

### High precision predictions under various culture conditions

**Figure 5:**
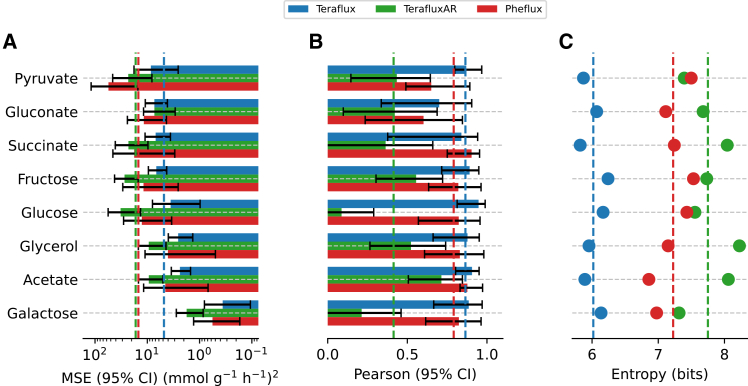
Model performance and entropy analysis for different culture conditions (A) shows the MSEs between experimental and estimated metabolic fluxes by Teraflux (blue), the unconstrained control model TerafluxAR (green), and Pheflux (red), under different culture conditions. Likewise, (B) shows the Pearson correlation coefficients or these models. (C) depicts the entropy values calculated for the same metabolic fluxes. The dashed lines represent the average values across conditions for each model. The culture conditions evaluated vary in the limiting carbon source used, namely: glucose, fructose, succinate, acetate, galactose, glycerol, gluconate, and pyruvate. Confidence intervals (CIs) were computed using a bootstrap procedure (*n* = 1,000).

We next evaluated Teraflux’s ability to simulate cellular metabolism using real transcriptomic data. We utilized gene expression profiles and exchange fluxes (glucose and biomass) from Gerosa et al.,^47^ who cultured *E. coli* under eight different conditions, using their ^13^C-derived fluxes as a benchmark. For comparison, we also evaluated Pheflux, a maximum entropy-based method previously reported to outperform other transcriptome-aware constraint-based models.^40^ Teraflux demonstrated strong performance, achieving an average mean squared error (MSE) of 4.802 (mmol g^−1^ h^−1^)^2^, notably lower than the 14.82 obtained by Pheflux (Figure 5A). Furthermore, Teraflux achieved a Pearson correlation of 0.865 (Figure 5B), slightly surpassing the 0.791 correlation observed for Pheflux. We observed consistent results across eight additional benchmarks spanning three organisms: *Bacillus subtilis* (five conditions), *Scheffersomyces stipitis* (one condition), and *Saccharomyces cerevisiae* (two conditions). As shown in Figure S1, Teraflux achieved the lowest average MSE across these benchmarks (17.40 vs. 32.89 (mmol g^−1^ h^−1^)^2^ for Pheflux). Similarly, Teraflux obtained a higher average Pearson correlation (0.8028) compared to Pheflux (0.7371).

To explicitly test the contribution of the thermodynamic irreversibility constraints (*v* ≥ 0) to this predictive accuracy, we evaluated a control version of our method where all reactions were treated as reversible (TerafluxAR). Removing these inequality constraints resulted in a substantial deterioration of predictive performance. TerafluxAR yielded a significantly higher MSE and a sharply lower Pearson correlation with experimental ^13^C-flux data compared to the fully constrained Teraflux model (Figures 5A and 5B). These results demonstrate that gene expression data and stoichiometric mass balance (*Sv* = 0) are, by themselves, insufficient to fully determine the fluxome. The inequality constraints act as critical, active boundary conditions derived from biologically meaningful kinetic priors. The degradation in performance upon removing them confirms that they restrict the solution space to a biologically meaningful subspace.

Metabolic networks typically follow a power-law distribution,^25^ where a few high-magnitude fluxes can artificially inflate Pearson correlation coefficients, potentially masking poor performance in lower-flux pathways. To address this bias, we also evaluated performance using the inverse hyperbolic sine (arcsinh) transformation. This transformation compresses the dynamic range of the data (see Figures S2–S5), ensuring that the correlation metric reflects the model’s ability to predict the global flux distribution rather than just dominant reactions. This analysis revealed that on transformed *E. coli* data, Pheflux yielded an average Pearson correlation of 0.8565, outperforming Teraflux, which reached 0.8178 (see Figure S6). However, this trend was reversed across the other eight benchmarks (see Figure S7), where Teraflux achieved an average Pearson correlation of 0.8205 compared to 0.7502 for Pheflux.

Taken together across all 16 datasets, Teraflux outperformed Pheflux in both evaluation metrics. Specifically, Teraflux achieved a lower average MSE of 11.10 (mmol g^−1^ h^−1^)^2^ (0.8779 on arcsinh-transformed data) and a higher Pearson correlation of 0.8337 (0.8191 on arcsinh-transformed data). In contrast, Pheflux yielded a higher average MSE of 23.85 (mmol g^−1^ h^−1^)^2^ (1.081 on arcsinh-transformed data) and a lower Pearson correlation of 0.7641 (0.8033 on arcsinh-transformed data). These results indicate that while both methods provide robust predictions of the global flux distribution, Teraflux offers a significant improvement in prediction accuracy for high-magnitude, biologically dominant fluxes.

While both Pheflux and standard flux sampling can suffer from thermodynamically infeasible cycles, Pheflux mitigates this issue by maximizing flux entropy conditioned on gene expression data. This method produces more homogeneous flux distributions that tend to avoid extremely large, unrealistic fluxes within these cycles. However, we found that Teraflux generates fluxomes with a significantly lower average entropy than Pheflux (*p* < 0.05, *t* test) (Figure 5C). Fluxome entropy can be interpreted as the level of uncertainty about the state of the system.^25^ Therefore, the lower entropy of Teraflux’s solutions suggests a more constrained and certain prediction. This increased certainty comes from Teraflux’s core mechanism: it directly assigns zero flux to reactions that otherwise would be participating in thermodynamically infeasible cycles. By eliminating these artificial energy-generating loops, Teraflux concentrates flux onto the remaining, thermodynamically sound pathways, which cannot support the arbitrarily large fluxes seen in flux sampling. In short, by incorporating thermodynamic considerations, Teraflux reduces the observer’s uncertainty about the actual distribution of fluxes.

### Biologically relevant inferences from enforcing relaxed thermodynamic consistency

To assess the biological relevance of Teraflux’s fluxomes, we compared its predictions for central carbon metabolism in *E. coli* iJO1366 against those of Pheflux, focusing on the gluconate growth condition where Teraflux showed the biggest jump in prediction power (see Figure 5A). Under this condition, the analysis revealed Teraflux’s superior accuracy.

**Figure 6:**
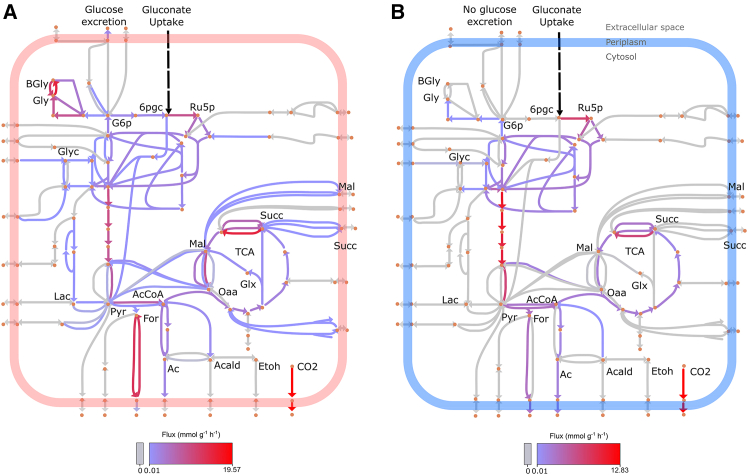
Thermodynamic consistency leads to meaningful inferences in the central carbon metabolism of *E. coli* iJO1366 (A) Estimation of metabolic fluxes using Pheflux showing excretion of glucose, cycles between glycogen (Gly) and branching glycogen (Bgly), and cycles between acetyl-CoA (AcCoA) and acetate (Ac), as well as recirculation between the cytoplasm and periplasm of TCA intermediates such as succinate (Succ) and malate (Mal). (B) Estimation of metabolic fluxes by Teraflux correctly predicting no glucose excretion, avoids infeasible cycles, and correctly predicts an inactive glyoxylate (Glx) shunt. Key molecules are highlighted, such as glycerol (Glyc), glucose 6-phosphate (G6p), 6-phospho-D-gluconate (6pgc), and ribulose 5-phosphate (Ru5p). The color of the lines represents the magnitude of the metabolic fluxes, with red indicating higher flux values and gray indicating no flux.

Unlike Pheflux (Figure 6A), Teraflux correctly predicted no glucose production or excretion and an inactive glyoxylate (Glx) shunt (Figure 6B), results that align with experimental observations for this condition.^47^ Teraflux also successfully avoided predicting the nonsensical recirculation of malate (Mal) and succinate (Succ) between the cytoplasm and the periplasm. This demonstrates an improvement in biological accuracy, as Pheflux incorrectly predicts these infeasible loops and an active glyoxylate shunt.

The advantages of Teraflux were also evident in other key pathways. For glycogen (Gly) metabolism, it accurately predicted the cycle to be inactive, which is consistent with the established biological principle that *E. coli* only synthesizes glycogen when carbon is abundant but another nutrient is limiting.^48^ Additionally, the iJO1366 model presents an interconversion loop between glycogen (Gly) and branching glycogen (BGly): BGly *⇌* Gly; where neither of the one-way reactions is coupled to energy-dissipating cosubstrates (e.g., ATP). As a result, both reactions being active would result in a thermodynamically infeasible cycle, an artifact that is prevented by Teraflux but not by Pheflux. Similarly, while investigating the conversion of acetyl-CoA (AcCoA) to acetate (Ac), Teraflux prevented a thermodynamically infeasible cycle by correctly avoiding a reverse flux from acetate back to acetyl-CoA. These examples underscore how generating flux distributions free of infeasible cycles leads to more biologically meaningful predictions.

### Teraflux fluxome and Δ*G* predictions are largely consistent with physicochemical constraints

**Figure 7:**
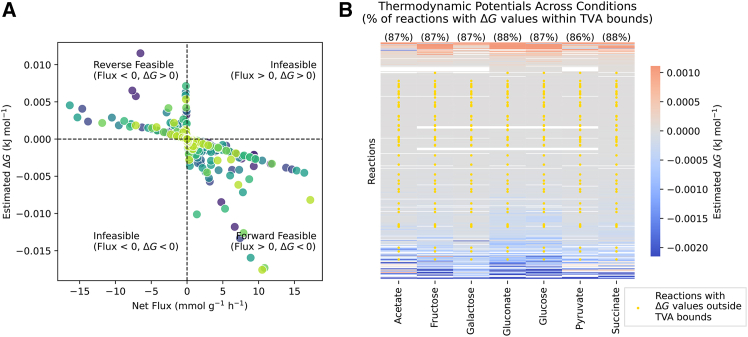
Thermodynamic consistency analysis of Teraflux-predicted metabolic states (A) Scatterplot of active net reaction fluxes (|*v*| > 1^−6^ mmol g^−1^ h^−1^) versus estimated Gibbs free energy changes (Δ*G*) across eight limiting carbon sources (acetate, fructose, galactose, gluconate, glucose, glycerol, pyruvate, and succinate) color-coded from dark purple (acetate) to light green (succinate). Under all conditions, Teraflux estimated net flux directions are consistent with their thermodynamic gradient: top-left (reverse flux, positive Δ*G*) and bottom-right (forward flux, negative Δ*G*). No points were found in the top-right and bottom-left quadrants representing inconsistencies where flux opposes the thermodynamic gradient. (B) Heatmap of estimated Δ*G* values for internal active metabolic reactions across seven carbon source conditions. Reactions are sorted by their mean Δ*G* value. The color scale indicates Gibbs free energy change values. Yellow markers highlight reactions where the Teraflux-estimated Δ*G* falls outside of the feasible range [Δ*G*_min_, Δ*G*_max_] derived from TVA. The percentage of reactions consistent with TVA bounds is shown above each column. Extracellular transport reactions were excluded from this analysis. To enhance visual contrast, the heatmap color scale limits were bound to the 2nd and 98th percentiles of the estimated Δ*G*.

One of the central features of Teraflux is the use of gene expression levels to estimate condition-specific fluxes and chemical potentials, *μ*, and, consequently, reaction Gibbs free energy changes, Δ*G* = *S*^*T*^*μ*. The Teraflux formulation ensures that reactions always proceed down the chemical potential gradient. We empirically validated this using results from *E. coli* iJO1366, where the net fluxes of active reactions (|*v*_*f*_ − *v*_*r*_| > 10^−6^) consistently maintain a sign opposite to their corresponding estimated Δ*G* values (Figure 7A). However, directional consistency alone does not guarantee that the inferred fluxes and Δ*G* magnitudes are realistic. To thoroughly assess whether these predictions are biologically meaningful, we employed a two-part validation using thermodynamic variability analysis (TVA).^9^ First, we evaluated if the inferred flux directions could be supported by any set of Δ*G* values consistent with physiological bounds on standard biochemical Gibbs free energies of reaction and intracellular metabolite concentrations. Second, we tested whether the specific Δ*G* values inferred by Teraflux fall within these physiologically realistic bounds.

Since actual Δ*G* values vary under different conditions as a function of intracellular metabolite concentrations—defined by Δ*G* = Δ*G*′^◦^ + *RT* ln *Q* (where Δ*G*′^◦^ is the standard biochemical Gibbs free energy of reaction and *Q* is the condition-specific ratio of product to reactant concentrations)—we computed their minimum and maximum possible ranges across the eight growth conditions for the *E. coli* iJO1366 model. For each condition, we formulated a linear optimization problem (see STAR Methods, thermodynamic variability analysis), where the decision variables are the standard biochemical Gibbs free energies of reaction and the natural logarithms of metabolite concentrations, to estimate the feasible range [Δ*G*_min_, Δ*G*_max_] for every internal reaction. This problem was subjected to three physicochemical constraints: (1) standard biochemical Gibbs free energies of reaction estimated via component contribution^49^ (adjusted to pH 7.0, *I* = 0.15M, and within 99% CI of mean Δ*G*′^◦^); (2) intracellular metabolite concentrations bounded within physiological ranges (1 μM to 100 mM) as previously reported for *E. coli*^50^; and (3) the application of the second law of thermodynamics, which requires that the Δ*G* values must have the opposite sign to the net fluxes predicted by Teraflux.

Addressing the first part of our validation, solving these condition-specific TVA problems confirmed that the reaction directions generated by the gene-expression weighting in Teraflux are largely physicochemically feasible. With the exception of the glycerol growth condition, for seven of the eight carbon sources tested, the solver successfully identified a consistent set of Δ*G* values and concentrations that satisfied all physicochemical constraints simultaneously. This confirms that the flux directions derived from the transcriptomic data can generally be supported by realistic physiological constraints.

To investigate whether the irreversibility constraints imposed by Teraflux (*v*_*i*_ ≥ 0, *∀i* ∈ *I*) were the root cause of the TVA infeasible solution observed in the glycerol condition, we performed the same validation on flux distributions generated by our baseline control model, TerafluxAR. As previously introduced, TerafluxAR treats all internal reactions as fully reversible. In stark contrast to the results obtained with the fully constrained Teraflux formulation, the fluxomes produced by TerafluxAR proved to be infeasible across all eight conditions. Without the irreversibility constraints, the solver could not find any set of physiological metabolite concentrations capable of supporting the predicted flux directions. This failure at the first step of our validation indicates widespread violations of the physicochemical constraints imposed by TVA. These results demonstrate that the irreversibility constraints do not over-constrain the glycerol condition into infeasibility. Rather, for this specific outlier, transcriptomics and basic directional priors alone are simply insufficient to guide the flux distribution into a fully physicochemically consistent state.

Having established the feasibility of Teraflux flux directions for most conditions, the second part of our validation assessed the consistency of its inferred Δ*G* with physiological constraints. As described in the supplemental information Methods S1, Teraflux can estimate *μ* as −2*RT*(*S*^*T*^*λ*∗), where $\lambda^{*}\in R^{M}$ are the mass-balance associated Lagrange multiplier values at the optimal solution. When comparing the inferred Δ*G* = *S*^*T*^*μ* with physiologically constrained TVA bounds, we observed a high degree of quantitative agreement. As shown in Figure 7B, 86% (pyruvate) to 88% (gluconate and succinate) of the specific Δ*G* values derived from Teraflux’s Lagrange multipliers fell strictly within the feasible ranges [Δ*G*_min_, Δ*G*_max_] predicted by TVA. Within the Teraflux framework, Δ*G* can equivalently be expressed as a function of the unidirectional fluxes of active reactions, Δ*G* = *RT* ln(*v*_*r*_/*v*_*f*_) (see Equation 11). Based on this, we hypothesize that for the remaining 12%–14% of reactions that fell outside the TVA bounds, the inferred net flux directions are correct, but the specific magnitudes of their forward and reverse components (*v*_*f*_ and *v*_*r*_) yield natural logarithm ratios outside the physicochemical constraints imposed by TVA.

While both the flux directions and the specific Δ*G* predictions of Teraflux proved physicochemically consistent for the vast majority of conditions, the infeasibility of the glycerol condition, along with the 12%–14% of specific Δ*G* estimates lying outside the TVA ranges in the other seven cases, highlight the method’s boundaries. This discrepancy indicates that while transcriptomics, in general, can reliably specify flux directionality and most forward and reverse fluxes, additional information is required for specific outliers to fully align flux magnitudes with realistic cellular physicochemical constraints.

## Discussion

The accurate estimation of metabolic fluxes is crucial for understanding cellular metabolism and developing effective metabolic engineering strategies. Traditional constraint-based methods often suffer from the presence of thermodynamically infeasible cycles, which can lead to unrealistic and inaccurate flux predictions. To address this, we have developed Teraflux, a method that integrates the principle of maximum entropy with transcriptomic data to generate condition-specific fluxome estimations that are free of such cycles.

Teraflux offers several advantages over existing methods. First, by maximizing entropy within a thermodynamically informed framework, it ensures that the resulting flux distributions are minimally biased and avoid infeasible loops. Second, it incorporates transcriptomic data, allowing for the prediction of condition-specific fluxomes that reflect underlying gene expression patterns. Third, it includes constraints that impose the irreversibility of reactions, ensuring that predictions align with prior biological knowledge on reaction kinetics. Finally, Teraflux processes genome-scale metabolic models, such as iJO1366, within seconds and uses less than 2 Gb of RAM.

Beyond these practical advantages, Teraflux rests on a distinct theoretical foundation. While sharing similarities with the variational method of Fleming et al.,^26^ our approach is framed from an information-theoretic perspective, resulting in an optimization problem that minimizes the Kullback-Leibler divergence between a gene-expression-based probability distribution and a fluxome-based one. This framework formally identifies 1 − ln(*g*) as the correct weighting factor for incorporating transcriptomics. Furthermore, analysis of the Karush-Kuhn-Tucker conditions reveals that the dual variable associated with the *v* ≥ 0 constraint serves as a measure for unspent thermodynamic driving force for a kinetically blocked reaction. This approach seamlessly bridges thermodynamics and kinetics: it acknowledges that chemical potentials dictate a reaction’s direction rather than its absolute rate, using flux constraints as a proxy for kinetic barriers without improperly overfitting the dual space of global chemical potentials. This allows the model to predict zero flux for such effectively irreversible reactions that would otherwise predict flux in the kinetically forbidden direction.

We have demonstrated the effectiveness of Teraflux on a variety of metabolic networks, showing that it can accurately predict metabolic fluxes while avoiding thermodynamically infeasible cycles. Crucially, as validated through TVA, the flux directions and the majority of the specific Gibbs free energy changes inherently predicted by the model align strictly with rigorous physiological limits on intracellular metabolite concentrations. It correctly preserves feasible cycles like the glyoxylate shunt, which are often missed by loopless methods based on the minimization of fluxes’ magnitudes, and demonstrates strong performance in predicting metabolic fluxes under various culture conditions using real transcriptomic data.

In conclusion, Teraflux is a valuable tool for studying metabolism. By maximizing entropy while enforcing a relaxed thermodynamic consistency sufficient to eliminate infeasible cycles, and producing condition-specific fluxomes grounded in physiological reality, Teraflux provides a more realistic and comprehensive view of cellular metabolism. This information can be used to design more effective metabolic engineering strategies and gain a deeper understanding of cellular biochemistry.

### Limitations of the study

Despite its strengths, Teraflux has several limitations. As evidenced by the thermodynamic infeasibility of the glycerol condition and the 12%–14% of specific Δ*G* estimates falling outside TVA bounds in our other tested environments, relying solely on transcriptomic data to dictate unidirectional flux ratios (*v*_*r*_/*v*_*f*_) can occasionally over-constrain flux magnitudes beyond what actual metabolite pools can thermodynamically support. The model’s accuracy is dependent on the quality of input transcriptomic data and fundamentally assumes that gene expression is a direct proxy for enzyme activity, which can be confounded by post-translational modifications and allosteric regulation. Additionally, Teraflux applies the same gene expression constraint symmetrically to both forward and reverse fluxes, a simplification that may not reflect independent biological regulation. By constraining the net fluxes of irreversible reactions based on prior kinetic knowledge, the model can also predict zero flux for a reaction even when a non-zero thermodynamic driving force exists, potentially masking as inactive fluxes that, in reality, exist but are undetectable by current measuring devices. Furthermore, our framework implicitly assumes that biochemical transformations occur in a homogeneous solution. Consequently, predictions may be inaccurate for reactions that become thermodynamically feasible only within non-homogeneous local environments where metabolite concentrations differ from the bulk. Moreover, while the lower MSE value achieved by Teraflux compared to Pheflux on experimental benchmarks is encouraging, these measurements capture only a fraction of all the reactions in a metabolic network. Hence, further validation is advisable if more flux measurements become available. Finally, as a steady-state method, Teraflux cannot capture metabolic dynamics over time; integrating it with dynamic frameworks, however, presents an interesting direction for future research.

## Resource availability

### Lead contact

Further information and requests for resources and reagents should be directed to and will be fulfilled by the lead contact, Marcelo Rivas-Astroza (marcelo.rivas@utem.cl).

### Materials availability

This study did not generate new unique reagents.

### Data and code availability

• The transcriptomic and fluxomic data analyzed in this study are derived from previously published studies. The sources are listed in the key resources table.
• The computational implementation of Teraflux as a Python library, as well as all code used to generate the results of this manuscript, are publicly available at: https://github.com/mrivas/teraflux. This link is also listed in the key resources table.
• Any additional information required to reanalyze the data reported in this paper is available from the lead contact upon request.

## Acknowledgments

This work was supported by the 10.13039/501100020884Agencia Nacional de Investigación y Desarrollo via 10.13039/501100002850FONDECYT Iniciación 11241181 ETAPA 2025 and FOVI 230173; the Cluster Faraday UTEM [CONICYT-FONDEQUIP—EQM180180]; and the Competition for Research Regular Projects, year 2022, code LPR22-07, Universidad Tecnológica Metropolitana.

## Author contributions

Authors contributed as follows. N.A.-T. and M.F.-M., conceptualization, data curation, formal analysis, investigation, software, validation, visualization, and writing – review and editing; D.T. and R.C., conceptualization, formal analysis, investigation, supervision, and writing – review and editing; M.R.-A., conceptualization, data curation, formal analysis, funding acquisition, investigation, project administration, software, supervision, validation, visualization, writing – original draft, and writing – review and editing.

## Declaration of interests

The authors declare no competing interests.

## Declaration of generative AI and AI-assisted technologies in the writing process

During the preparation of this work, the authors used Gemini (Google) in order to refine scientific writing, improve clarity and logical flow, and assist in editing for grammar and style. After using this tool, the authors reviewed and edited the content as needed and take full responsibility for the content of the publication.

## STAR★Methods

### Key resources table

**Table:** 

REAGENT or RESOURCE	SOURCE	IDENTIFIER
**Deposited data**		

*S. cerevisiae* genome-scale metabolic network	Mo et al.^51^	iMM904
*S. cerevisiae* RNA-seq transcriptomics	Nookaew et al.^52^	Chemostat and batch, using glucose as carbon source
*S. cerevisiae*^13^C fluxomics	Papini et al.^53^	Chemostat and batch, using glucose as carbon source
*S. stipitis* genome-scale metabolic network	Liu et al.^54^	iTL885
*S. stipitis* RNA-seq transcriptomics	Papini et al.^53^	Chemostat and glucose as carbon source
*S. stipitis*^13^C fluxomics	Papini et al.^53^	Chemostat and glucose as carbon source
*E. coli* genome-scale metabolic network	Orth et al.^1^	iJO1366
*E. coli* microarray transcriptomic	Gerosa et al.^47^	Eight different carbon sources
*E. coli*^13^C fluxomics	Gerosa et al.^47^	Eight different carbon sources
*B. subtilis* genome-scale metabolic network	Oh et al.^1^	iYO844
*B. subtilis* microarray transcriptomics	Nicolas et al.^55^	Five different carbon sources.
*B. subtilis*^13^C fluxomics	Chubukov et al.^56^	Five different carbon sources.

**Software and algorithms**		

Teraflux (v 1.0.1)	This paper	https://github.com/mrivas/teraflux

Table 1: key resources table

### Method details

#### Metabolic network modeling

A metabolic network represents the complete set of biochemical reactions and transport processes occurring within an organism. We employ a mathematical framework based on stoichiometry to model these networks, considering the conservation of mass.

Consider a network with *M* metabolites and *N* reactions, which includes internal biochemical reactions and exchange reactions for transport across the system boundary. The rates of the internal reactions are quantified by the internal flux vector $v\in R^{N}$, where each element represents the net flux of a reaction in units of mmol g^−1^ h^−1^. Each flux can be constrained by lower (*LB*) and upper (*UB*) bounds to encode reaction directionality or known flux rates. Internal reactions with unknown direction are treated as reversible, and their net flux *v* is expressed as the difference between a forward flux component $\left(v_{f}\in R^{N}\right)$ and a reverse flux component $\left(v_{r}\in R^{N}\right)$, both positive real numbers. Thus, the net internal flux vector is *v* = *v*_*f*_ − *v*_*r*_.

The stoichiometry of the internal reactions is captured by the stoichiometric matrix $S\in R^{M\times N}$, where each element *S*_*ij*_ denotes the stoichiometric coefficient of metabolite *i* in internal reaction *j*. Additionally, metabolic networks exchange mass with their environment through exchange fluxes, which are described by the stoichiometric matrix $S_{e}\in R^{M\times K}$ and the corresponding set of exchange fluxes $v_{e}\in R^{K}$. The principle of mass conservation dictates that the change in concentration $\left(c\in R^{M}\right)$ of each metabolite over time $\left(\dot{c}\right)$ is determined by the net sum of fluxes producing or consuming it. This dynamic relationship is expressed as:

(Equation 1) $$\dot{c}=S\left(v_{f}-v_{r}\right)+S_{e}v_{e}$$

In many metabolic modeling applications, particularly when analyzing specific phenotypes assumed to be stable, the system is considered to be at a steady state, meaning the concentrations of internal metabolites are constant $\left(\dot{c}=0\right)$. Under this assumption, the mass balance equation simplifies to:

(Equation 2) $$S\left(v_{f}-v_{r}\right)+S_{e}v_{e}=0$$

This equation defines the space of flux distributions that are consistent with mass conservation for a given network structure and exchange fluxes. However, satisfying mass balance alone is insufficient to guarantee biological realism. The solution space permitted by stoichiometry can include thermodynamically infeasible flux distributions, such as internal metabolic cycles or loops where metabolites are perpetually interconverted without net input or output, akin to a perpetual motion machine. To estimate a fluxome that is uniquely determined and consistent with the second law of thermodynamics, further considerations must be taken into account.

#### Thermodynamic consistency via variational principle

In order to be consistent with the second law of thermodynamics, the net fluxes in a metabolic network must always proceed down a thermodynamic gradient. Formally, for the chemical potentials of each metabolite in the network, $\mu \in R^{M}$, the Gibbs free energy differences over each reaction, Δ*G* = *S*^*T*^*μ*, must satisfy:

(Equation 3) $$\left(v_{f}-v_{r}\right)\cdot \Delta G\leq 0$$

where the equality is met if and only if Δ*G* = 0 and (*v*_*f*_ − *v*_*r*_) = 0.^6^^,^^57^

If we assume a chemical system is well-mixed, kept at a constant temperature and pressure, and its reactions follow mass-action rules, then we can write an equation that defines the Gibbs free energy difference using the rates of the forward and backward reactions.^58^

(Equation 4) $$\Delta G=RT\log\left(\frac{v_{r}}{v_{f}}\right),$$

which matches the strict thermodynamic constraints of Equation 3. In other words, strict thermodynamic consistency implies net zero fluxes are only possible if the corresponding Gibbs free energy difference is zero, and vice versa.^6^ A relaxed version of these constraints, as described and employed in,^20^^,^^21^^,^^24^^,^^57^^,^^59^ dispenses with the if and only if condition by allowing zero net fluxes even in the presence of non-zero Gibbs free energy differences. While this is at odds with the underlying assumptions sustaining Equation 4, this provides a practical solution to incorporating biological information that some reactions occur so slowly that their rate is effectively zero even when the thermodynamic driving force may not be.

To find a state that upholds the strict thermodynamic consistency, Fleming et al. proposed the following convex optimization problem derived from a variational principle^26^:

(Equation 5) $$\underset{v_{f},v_{r}}{\max}\;-v_{f}^{T}\left(\log v_{f}+k-o\right)-v_{r}^{T}\left(\log v_{r}+k-o\right)$$

(Equation 6) $$\text{subject}\;\text{to:}\;S\left(v_{f}-v_{r}\right)+S_{e}v_{e}=0$$

where $k\in R^{N}$ is a vector of adjustable parameters and $o\in R^{N}$ a vector of ones. The optimality conditions of this problem ensure that Equation 4 holds, which precludes thermodynamically infeasible cycles by assigning a unique chemical potential to each metabolite. However, the free selection of the parameter *k*, combined with the requirement that all reactions be treated as reversible, can assign large, reverse flux values to reactions known for kinetic reasons to be effectively irreversible.

#### Phenotype specificity via maximum entropy and transcriptomics

To estimate a single fluxome reflecting a specific cellular state, our group previously developed Pheflux.^40^ This method integrates transcriptomic data assuming gene expression levels are proxies for the abundance of the enzyme complexes catalyzing each reaction. This approach uses an information-theoretic perspective where fluxes are normalized to represent the probability of a reaction event occurring within the total metabolic activity. Pheflux then maximizes the Shannon entropy of the flux per mRNA unit across the network:

(Equation 7) $$H_{g}\left(v\right)=-v_{f}^{T}\log\frac{v_{f}}{g}-v_{r}^{T}\log\frac{v_{r}}{g}$$

subject to mass balance and other constraints. The vector $g\in R^{N}$ contains the enzyme complex transcriptome expression level for each reaction. Each value is computed by applying a gene-protein-reaction (GPR) rule to its associated gene expression data. The gene data itself is normalized to account for factors like library size and gene length, with Transcripts Per Million (TPM) being a common example for RNA-seq, and quantile normalization for micro-arrays. Pheflux makes the simplifying assumption that gene expression values weigh the forward and reverse fluxes of a reaction equally.

Maximizing Equation 7 is equivalent to minimizing the Kullback-Leibler divergence between the flux distribution and the transcriptomic distribution, yielding the unique, least biased flux distribution consistent with the observed transcriptome. However, in its original formulation, Pheflux splits only reactions designated as reversible based on prior knowledge; effectively irreversible reactions are represented by a single variable. While this incorporates knowledge of reaction directionality, it removes the guarantee that all thermodynamically infeasible cycles will be eliminated.

#### Unified framework: Teraflux

We propose Teraflux, a unified method that is phenotype-specific and incorporates prior kinetic knowledge about reaction directionalities, while computing a fluxome consistent with the relaxed thermodynamic constraints (and therefore free of infeasible cycles). We establish an equivalence between the objective functions of the thermodynamically-focused approach of Fleming et al.^26^ and the phenotype-specific approach of González-Arrué et al*.*^40^ The objective function of Pheflux can be written in a form that is directly comparable to that of Fleming et al. if their parameter vector *k* is chosen such that *k* − *o* = − log  *g*.

Leveraging this equivalence, Teraflux utilizes the structure of Fleming et al.’s framework, explicitly modeling forward (*v*_*f*_) and reverse (*v*_*r*_) fluxes for all reactions, which is foundational for addressing thermodynamic feasibility. Phenotype specificity is incorporated by setting the parameters based on transcriptomic data *g* resulting in a framework that is equivalent to minimize the Kullback-Leibler divergence between the fluxome and the transcriptome. To address the kinetic limitations of irreversible reactions, which are not fully accounted for by thermodynamic arguments alone, Teraflux adds inequality constraints for net reaction fluxes. From a biophysical perspective, this distinction is crucial because chemical affinity dictates only the direction of a reaction, not its absolute rate. A highly negative Δ*G* implies that if the reaction occurs, *v*_*f*_ ≫ *v*_*r*_, but it does not preclude *v* ≈ 0 due to kinetic barriers such as enzyme availability, allosteric regulation, or spatial sequestration. Constraining the fluxes acts as a parsimonious proxy for these complex, unmodeled physical constraints, without improperly forcing the chemical potentials to explain local kinetic limitations. Furthermore, from an information-theoretic standpoint, this approach corresponds to incorporating prior partial knowledge into the Maximum Entropy framework. By applying the inequality constraint *v*_*i*_ ≥ 0, we restrict the flux search space to physically realizable values. Maximizing entropy within this restricted space avoids the over-fitting that would occur if we attempted to constrain the chemical potentials directly without explicit concentration measurements. This renders Teraflux as the following optimization problem:

(Equation 8) $$\underset{v_{f},v_{r}}{\max}\;-v_{f}^{T}\left(\log v_{f}-\log g\right)-v_{r}^{T}\left(\log v_{r}-\log g\right)$$

(Equation 9) $$\text{subject}\;\text{to:}\;S\left(v_{f}-v_{r}\right)+S_{e}v_{e}=0$$

(Equation 10) $$v_{f,i}-v_{r,i}\geq 0\;\forall i\in I$$

where *I* is the set of reactions known to be irreversible.

The addition of inequality constraints means that strict thermodynamic consistency is not guaranteed because the optimality conditions now include dual variables $\alpha \in R^{N}$ associated with the irreversibility constraints, resulting in the following, modified thermodynamic relationship:

(Equation 11) $$RT\log\left(\frac{v_{r}}{v_{f}}\right)=\Delta G+2RT\alpha$$

However, as we demonstrate in the Supplementary Material (see Methods S1), the dual variables *α* are zero for all reversible reactions and any irreversible reactions carrying non-zero net flux, while they can be non-zero for an irreversible reaction with zero net flux. This implies a relaxing of the strict application of the Second Law of Thermodynamics, but one that still ensures reactions always proceed in the direction compatible with their Gibbs free energy difference, unless prohibited from doing so based on kinetic limitations. In this case, *α* effectively quantifies the Gibbs free energy difference not exploited by a kinetically blocked reaction, and this set of relaxed conditions is related to but still slightly stronger than those considered in.^20^^,^^21^^,^^24^^,^^57^^,^^59^ In particular, these conditions are still sufficient to exclude the existence of thermodynamically infeasible cycles, which we also show (see supplementary information Methods S1) is not the case for Pheflux.

### Quantification and statistical analysis

#### Thermodynamic variability analysis

The validation of the fluxes directions and estimated Δ*G* values was conducted on the *E. coli* iJO1366 genome-scale metabolic network, chosen for its high-quality annotation and compatibility with the equilibrator-api framework^49^ for standard energy estimation. For each of the eight experimental growth conditions, we formulated a linear optimization problem to estimate the feasible thermodynamic range [Δ*G*_min_, Δ*G*_max_] for every internal reaction in the network, subject to the flux directions predicted by Teraflux. The continuous decision variables in this linear program are the standard biochemical Gibbs free energies of reaction $\left(\Delta_{r}G_{j}^{\prime ◦}\right)$ and the natural logarithms of the metabolite concentrations (ln *c*_*i*_).

The optimization problem for each reaction *k* is defined as follows:

(Equation 12) $$\begin{array}{ll} \text{minimize/maximize}\; & \Delta G_{k}\\ \text{subject}\;\text{to:}\; & \\ \; & \Delta G_{j}=\left(\Delta_{r}G_{j}^{\prime ◦}\right)+RT\underset{i}{\sum }S_{ij}\ln c_{i},\;\forall j \end{array}$$

(Equation 13) $$\gamma_{j}-2.58\sigma_{j}\leq \left(\Delta_{r}G_{j}^{\prime ◦}\right)\leq \gamma_{j}+2.58\sigma_{j},\;\forall j$$

(Equation 14) $$\ln\left(10^{-6}\right)\leq \ln c_{i}\leq \ln\left(10^{-1}\right),\;\forall i$$

(Equation 15) $$\text{sign}\left(v_{j}^{\text{teraflux}}\right)\cdot \Delta G_{j}\leq -\epsilon ,\;\forall j\in R_{\text{active}}$$

where.

• Standard energies (Equation 12): The standard biochemical Gibbs free energies of reaction $\left(\Delta_{r}G_{j}^{\prime ◦}\right)$ were bounded using the Component Contribution method via equilibrator-api^49^ (adjusted to pH 7.0, I = 0.15M). We constrained these values to lie within a 99% confidence interval (2.58*σ*_*j*_) of the estimated mean *γ*_*j*_ (Equation 13).
• Physiological constraints (Equation 14): Intracellular metabolite concentrations *c*_*i*_ were bounded within the broad physiological range of 1 *μM* to 100 *mM*.^50^
• Flux-Force coupling (Equation 15): For all reactions where Teraflux predicted a significant active net flux $\left(v_{j}^{\text{teraflux}}\right)$, we imposed the Second Law of Thermodynamics, ensuring that the net flux flows down the thermodynamic gradient (where *ϵ* represents a minimum driving force of 10^−3^ kJ/mol).

#### Computational implementation

Teraflux was implemented as a strictly convex non-linear optimization problem using iPOPT (Interior Point OPTimizer) in conjunction with the CasADi library.^60^^,^^61^ For Loopless Flux Variability Analysis and Flux Sampling analyses, the GLPK (GNU Linear Programming Kit) solver was used.^62^ The integration of these components was carried out in the Python programming environment, using the COBRApy library.^45^^,^^46^ All computations were run in an Ubuntu 22.04.5 server with a 13th Gen Intel Core i7-13700KF (32 threads) and 32 Gb of RAM.

#### Data source for benchmarking

To benchmark Teraflux, we used two distinct experimental datasets from *E. coli*. For the predicted fluxes in the glyoxylate shunt, we used ^13^C-derived flux data from Ishii et al.^41^ corresponding to a growth rate of 0.1 h^−1^. For broader validation, we used a dataset from Gerosa et al.^47^ which provided experimental fluxes, metabolite data, and gene expression patterns for *E. coli* K12.

These datasets were integrated with the iJO1366 metabolic model^1^ to run the simulations. The resulting flux distributions for the central carbon system were then visualized with the Escher web application.^63^

We also validated our method using data from three additional microorganisms grown under 8 conditions, as previously published.^52^^,^^53^^,^^55^^,^^56^ This dataset includes matched transcriptomic (RNA-seq or microarray) and ^13^C-flux data. We used the specific genome-scale metabolic models for each species. Exchange constraints were applied to mimic the nutrient uptake rates and the biomass growth rate as described in the original publications. The details of the full dataset can be seen in the key resources table.

#### Bioinformatic analysis

RNA-seq libraries for *S. cerevisiae* and *S. stipitis* (see key resources table) were mapped to their respective reference assemblies (sacCer3, CBS 6054) using STAR 2.5.0a^64^ with default parameters. Gene expression was quantified as Fragments Per Kilobase of exon per Million mapped reads (FPKM). For *E. coli* and *B. subtilis*, microarray data were processed using quantile normalization via the limma R package.^65^
