# Supplementary material for: Thermodynamically enabled and reaction attuned estimation of metabolic fluxes
Source: iScience. 2026 Apr 21;29(6):115822. doi: 10.1016/j.isci.2026.115822 (PMC13156759; doi:10.1016/j.isci.2026.115822)
Supplement: Document S1. Figures S1–S7 and Methods S1 [file mmc1.pdf]

iScience, Volume *29*

Supplemental information

Thermodynamically enabled and reaction attuned estimation of metabolic fluxes

Nicolás Améstica-Toledo, Maximiliano Farías-Miño, Raúl Conejeros, David Tourigny, and Marcelo Rivas-Astroza

1 Methods S1: Mathematical Proof of Relaxed Thermodynamic Consistency.

This supplementary material provides a mathematical proof demonstrating that the Teraflux optimization framework ensures the resulting flux estimations are consistent with prior knowledge of reaction directionality. Specifically, we show how the introduction of lower bounds on irreversible reactions maintains the core thermodynamic principles that prevent infeasible cycles, satisfying a condition termed relaxed thermodynamic consistency [1, 2].

1.1 Problem Formulation

For a metabolic network of M metabolites and N reactions, we consider the following optimization problem, aiming to find the forward (v_f) and reverse (v_r) reaction flux vectors:

$$\max_{v_f, v_r} \quad -v_f^T(\log v_f - \log g) - v_r^T(\log v_r - \log g) \tag{A.1}$$

$$\text{subject to:} \quad S(v_f - v_r) + S_e v_e = 0 \tag{A.2}$$

$$v_{f_i} - v_{r_i} \geq 0 \quad \forall i \in I. \tag{A.3}$$

In this formulation, the terms are defined as follows:

- $S \in \mathbb{R}^{M \times N}$ is the stoichiometric matrix.
- $S_e v_e \in \mathbb{R}^M$ represents external boundary fluxes, which are assumed to be fixed.
- $g \in \mathbb{R}^N_{>0}$ is a vector of strictly positive constants representing the gene expression level associated with each reaction's enzyme complex.
- I is the index set of reactions assumed to be effectively irreversible based on biological priors regarding reaction kinetics.

Moreover, as established by [3], we note that the strict inequality constraints $v_f, v_r > 0$ are implicitly enforced by the domain of the natural logarithm in the objective function.

1.2 Variational Framework and KKT Optimality Conditions

To formulate a variational framework for the problem defined by (A.1), (A.2), and (A.3), we construct a Lagrangian functional $\mathcal{L}$. We introduce dual variables $\lambda \in \mathbb{R}^M$ for the steady-state mass balance constraints (A.2) and $\alpha \in \mathbb{R}^N_{\geq 0}$ for the irreversibility constraints, with the understanding that $\alpha_i = 0$ for all unconstrained reversible reactions ($i \notin I$) and $\alpha_i \geq 0$ for all constrained irreversible reactions ($i \in I$). The full Lagrangian is therefore:

$$\begin{aligned}\mathcal{L}(v_f, v_r, \lambda, \alpha) = &- v_f^T(\log v_f - \log g) - v_r^T(\log v_r - \log g) \\ &+ \lambda^T[S(v_f - v_r) + S_e v_e] \\ &+ \alpha^T[v_f - v_r].\end{aligned} \tag{A.4}$$

According to the Karush-Kuhn-Tucker (KKT) conditions, correctly handling the inequality constraints requires the enforcement of complementary slackness:

$$\alpha_i(v_{f_i} - v_{r_i}) = 0 \quad \forall i \in I. \tag{A.5}$$

1.2.1 Stationarity Conditions for v_f and v_r

Taking the partial derivatives of the Lagrangian with respect to each component v_{f_i} and v_{r_i} and setting them to zero yields:

$$\frac{\partial \mathcal{L}}{\partial v_{f_i}} = -\log v_{f_i} + \log g_i - 1 + (S^T\lambda)_i + \alpha_i = 0 \tag{A.6}$$

$$\frac{\partial \mathcal{L}}{\partial v_{r_i}} = -\log v_{r_i} + \log g_i - 1 - (S^T\lambda)_i - \alpha_i = 0. \tag{A.7}$$

Subtracting Equation (A.7) from (A.6) gives:

$$\log v_{r_i} - \log v_{f_i} + 2(S^T\lambda)_i + 2\alpha_i = 0. \tag{A.8}$$

Rearranging this equation provides the fundamental relationship between the unidirectional flux ratio and the dual variables:

$$\log\left(\frac{v_{r_i}}{v_{f_i}}\right) = -2(S^T\lambda)_i - 2\alpha_i \tag{A.9}$$

where the vector operations are performed element-wise.

1.2.2 Complementary Slackness Conditions

While the dual variables λ are unconstrained in this variational formulation, and $\alpha_i = 0$ by construction for reversible reactions, the KKT conditions require α_i for irreversible reactions to satisfy:

$$\alpha_i \geq 0, \quad \alpha_i(v_{f_i} - v_{r_i}) = 0 \quad \forall i \in I. \tag{A.10}$$

These complementary slackness conditions, associated with the original inequality constraint (A.3), carry critical implications for the thermodynamic consistency of the optimal flux distribution. Physically, this dictates that if an irreversible reaction $i \in I$ carries a non-zero net flux in the optimal solution ($v_{f_i} - v_{r_i} > 0$), we must have $\alpha_i = 0$. However, the converse is not strictly guaranteed: if an irreversible reaction i carries exactly zero net flux ($v_{f_i} - v_{r_i} = 0$), the optimal solution permits either $\alpha_i > 0$ or $\alpha_i = 0$.

1.3 Connection to Thermodynamics and Interpretation of α

As originally described by [3], the derivations above provide an explicit theoretical link between the variational framework and the principles of thermodynamic consistency. In that study, the optimal values of the mass-balance dual variables λ^* are directly proportional to chemical potentials. If we define the chemical potential vector μ such that $\mu = -2RT\lambda^*$, where R is the ideal gas constant and T is temperature, Equation (A.9) becomes:

$$RT\log\left(\frac{v_{r_i}}{v_{f_i}}\right) = \Delta G_i - 2RT\alpha_i \tag{A.11}$$

where we have identified $\Delta G_i = (S^T\mu)_i$ as the Gibbs free energy change for reaction i. When reaction i is reversible, or when the net flux carried by an irreversible reaction i is non-zero, then

$\alpha_i = 0$ (as demonstrated in the previous section), recovering the well-established thermodynamic identity:

$$RT \log \left(\frac{v_{r_i}}{v_{f_i}} \right) = \Delta G_i. \tag{A.12}$$

Under these conditions, the optimal solution provides a set of values for Gibbs free energy changes that strictly adhere to the Second Law of Thermodynamics, dictating that the net flux for each reaction must flow down its chemical potential gradient.

A strict application of the Second Law dictates that the net flux through a reaction is zero if and only if its Gibbs free energy change is zero. However, Equation (A.11) reveals that this strict application holds true only for reversible reactions in our framework. For irreversible reactions carrying zero net flux, we cannot guarantee that $\alpha_i = 0$. Substituting $v_{r_i}/v_{f_i} = 1$ into Equation (A.11) yields:

$$\Delta G_i = 2RT\alpha_i \geq 0. \tag{A.13}$$

Consequently, for irreversible reactions carrying zero net flux, a zero Gibbs free energy change ($\Delta G_i = 0$) is not strictly guaranteed.

Instead, the formulation enforces a *relaxed* version of the Second Law. Under this relaxed consistency, the biconditional relationship between zero net flux and zero ΔG_i is decoupled, permitting reactions with zero net flux to maintain a non-zero ΔG_i. Importantly, it is straightforward to show mathematically that $\Delta G_i = 0$ still implies zero net flux. This represents a refined implementation of the relaxed conditions described in prior literature [2, 1, 4, 5, 6], as those studies impose no restrictions on the value of ΔG_i when the net flux is zero.

Biologically, these relaxed thermodynamic conditions are physically realistic. ΔG_i indicates the thermodynamic potential for spontaneous reactivity, but it does not dictate the actual kinetic rate. Numerous biological scenarios exist where a thermodynamic driving force is available, but the catalytic potential is absent (e.g., due to allosteric inhibition or a lack of enzyme expression), causing the reaction to proceed at an effectively negligible rate. Equation (A.11) precisely encodes this physiological reality: either an irreversible reaction carries non-zero net flux in the forward direction (meaning its free energy difference is strictly negative), or its net flux is zero, in which case either $\Delta G_i = 0$ or $\Delta G_i > 0$ depending on the state of α_i. Through Equation (A.13), it becomes evident that the term $2RT\alpha_i$ effectively quantifies the unspent thermodynamic driving force of a kinetically blocked reaction.

1.4 Prevention of Thermodynamically Infeasible Cycles in Teraflux

Building upon the thermodynamic relationships established above, we can now mathematically demonstrate that the relaxed thermodynamic consistency enforced by Teraflux strictly prevents the formation of thermodynamically infeasible cycles (TICs), also known as Type II loops. A TIC is defined as a closed cycle of internal reactions through which a net flux circulates continuously at steady state, without any net consumption or production of external metabolites.

Proof by Contradiction:

Assume there exists a closed cycle of reactions, defined by the index set $\mathcal{C}$, such that a net flux circulates through the loop. By defining the cycle's forward direction along the flow of the circulation, the net flux for every reaction $i \in \mathcal{C}$ must be strictly positive:

$$v_{net_i} = v_{f_i} - v_{r_i} > 0 \quad \forall i \in \mathcal{C}. \tag{A.14}$$

Because the net flux is strictly positive for all reactions in the cycle, the complementary slackness condition (Equation A.10) dictates that $\alpha_i = 0$ for all $i \in \mathcal{C}$, regardless of whether they are classified as reversible or effectively irreversible.

Consequently, Equation A.11 simplifies to the standard thermodynamic identity for all reactions participating in the cycle:

$$\Delta G_i = RT \log \left(\frac{v_{r_i}}{v_{f_i}} \right) \quad \forall i \in \mathcal{C}. \tag{A.15}$$

Since $v_{f_i} > v_{r_i} > 0$, it follows that the ratio $v_{r_i}/v_{f_i} < 1$. Because $\log(x) < 0$ for $0 < x < 1$, the Gibbs free energy change for each step in the cycle must be strictly negative:

$$\Delta G_i < 0 \quad \forall i \in \mathcal{C}. \tag{A.16}$$

However, as defined in our framework, the Gibbs free energy change of a reaction is the difference between the chemical potentials of its products and reactants, $\Delta G = S^T \mu$. Because μ is a state function (a scalar field), its value depends only on the state of the metabolite, not on the path taken. Therefore, the summation of potential differences around any closed path must vanish (analogous to Kirchhoff's loop law for potentials).

Mathematically, any mass-balanced closed cycle can be represented by a null space vector $c \in \mathbb{R}^N$, where $c_i > 0$ assigns the relative strictly positive flux magnitude for each reaction $i \in \mathcal{C}$ required to maintain the cycle, and $c_i = 0$ otherwise. By the definition of a mass-balanced cycle, $Sc = 0$.

Because $c_i > 0$ and $\Delta G_i < 0$ for all reactions in the cycle, the flux-weighted sum of the Gibbs free energy changes around the closed loop must be strictly negative:

$$\sum_{i \in \mathcal{C}} c_i \Delta G_i < 0. \tag{A.17}$$

Conversely, evaluating this same sum using the state function property yields:

$$\sum_{i \in \mathcal{C}} c_i \Delta G_i = c^T \Delta G = c^T (S^T \mu) = (Sc)^T \mu = 0^T \mu = 0. \tag{A.18}$$

The results in Equation A.17 ($\sum c_i \Delta G_i < 0$) and Equation A.18 ($\sum c_i \Delta G_i = 0$) present a direct mathematical contradiction. Therefore, the initial assumption must be false: no net flux can circulate through a closed loop under these constraints. This formally guarantees that Teraflux fluxomes are entirely free of TICs.

1.5 Pheflux Does Not Guarantee Thermodynamic Consistency or Prevent TICs

We now demonstrate that our previous Pheflux formulation [7] does not guarantee the same thermodynamic consistency as Teraflux. Instead of imposing inequality constraints on the net difference between forward and reverse variables, the Pheflux framework can be viewed as an alternative that assigns only a single forward variable to irreversible reactions. Pheflux solves the following optimization problem:

$$\max_{v_f^R, v_r^R, v_f^I} \quad -(v_f^R)^T(\log v_f^R - \log g^R) - (v_r^R)^T(\log v_r^R - \log g^R) - (v_f^I)^T(\log v_f^I - \log g^I) \tag{A.19}$$

$$\text{subject to:} \quad S_R(v_f^R - v_r^R) + S_I v_f^I + S_e v_e = 0 \tag{A.20}$$

where variables are partitioned into reversible (R) and irreversible (I) components, inducing the same partitioning on the gene expression data and the stoichiometric matrix. Without loss of generality, irreversible reactions are assumed to possess only forward (v_f^I) components.

As with Teraflux, we use a variational approach by introducing the Lagrangian $\mathcal{L}$ with Lagrange multipliers $\lambda \in \mathbb{R}^M$ for the mass balance constraints:

$$\begin{aligned}\mathcal{L}(v_f, v_r, \lambda) = &- (v_f^R)^T(\log v_f^R - \log g^R) - (v_r^R)^T(\log v_r^R - \log g^R) - (v_f^I)^T(\log v_f^I - \log g^I) \\ &+ \lambda^T[S_R(v_f^R - v_r^R) + S_I v_f^I + S_e v_e]\end{aligned} \tag{A.21}$$

noting that in this formulation, there are no dual variables α to handle inequality constraints. The stationarity conditions are again obtained by taking partial derivatives with respect to each flux variable and setting them to zero:

$$\frac{\partial \mathcal{L}}{\partial v_{f_i}^R} = -\log v_{f_i}^R + \log g_i^R - 1 + (S_R^T\lambda)_i = 0 \tag{A.22}$$

$$\frac{\partial \mathcal{L}}{\partial v_{r_i}^R} = -\log v_{r_i}^R + \log g_i^R - 1 - (S_R^T\lambda)_i = 0 \tag{A.23}$$

$$\frac{\partial \mathcal{L}}{\partial v_{f_i}^I} = -\log v_{f_i}^I + \log g_i^I - 1 + (S_I^T\lambda)_i = 0. \tag{A.24}$$

Subtracting Equation (A.23) from Equation (A.22) and rearranging yields the usual thermodynamic relationship for reversible reactions. By associating the optimal value λ^* with chemical potentials ($\mu = -2RT\lambda^*$) and the Gibbs free energy change ($\Delta G^R = S_R^T\mu$), we obtain:

$$RT \log\left(\frac{v_r^R}{v_f^R}\right) = \Delta G^R. \tag{A.25}$$

However, because irreversible reactions lack a corresponding reverse variable, we cannot perform the same subtraction. We can only use Equation (A.24) to derive the following relationship:

$$2RT \log\left(\frac{g^I}{v_f^I}\right) - 2RT = \Delta G^I, \tag{A.26}$$

where $\Delta G^I = S_I^T\mu$ represents the Gibbs free energy change of the irreversible reactions.

Because $v_f^I > 0$, all irreversible reactions in this model proceed with a net positive flux. Therefore, strict thermodynamic consistency requires that $\Delta G^I < 0$. Imposing this necessary constraint on Equation (A.26) and rearranging translates to:

$$\log\left(\frac{g^I}{v_f^I}\right) < 1, \tag{A.27}$$

which (since $g^I > 0$ and assuming log denotes the natural logarithm) is satisfied if and only if $v_f^I > g^I/e$, where e is Euler's number.

There is evidently no guarantee that this mathematical constraint will be satisfied across all irreversible reactions for any given set of measured enzyme expression values. Because the Pheflux optimization problem does not strictly bound the primal flux magnitudes to satisfy this condition,

the solver is free to select a flux magnitude where $v_f^I < g^I/e$ in order to satisfy global mass balance. Any violation of Equation (A.27) implies that the reaction is proceeding spontaneously against its thermodynamic gradient ($\Delta G^I > 0$ while $v_f^I > 0$).

This formally demonstrates that the Pheflux framework is theoretically insufficient for excluding thermodynamically infeasible cycles from its optimal flux distributions. Specifically, if an irreversible reaction in Pheflux takes on a positive ΔG^I, it can artificially compensate for the negative ΔG of other reactions within a cycle, allowing the loop sum to reach zero ($\sum \Delta G = 0$) even while a net flux continuously circulates through all steps ($\sum \Delta G < 0$ would no longer be strictly required). Consequently, Pheflux is inherently susceptible to generating TICs.

References

[1] Daniel A Beard, Eric Babson, Edward Curtis, and Hong Qian. Thermodynamic constraints for biochemical networks. *Journal of theoretical biology*, 228(3):327–333, 2004.

[2] Arne C Müller and Alexander Bockmayr. Fast thermodynamically constrained flux variability analysis. *Bioinformatics*, 29(7):903–909, 2013.

[3] Ronan MT Fleming, Christopher M Maes, Michael A Saunders, Yinyu Ye, and Bernhard Ø Palsson. A variational principle for computing nonequilibrium fluxes and potentials in genome-scale biochemical networks. *Journal of theoretical biology*, 292:71–77, 2012.

[4] Jan Schellenberger, Nathan E Lewis, and Bernhard Ø Palsson. Elimination of thermodynamically infeasible loops in steady-state metabolic models. *Biophysical journal*, 100(3):544–553, 2011.

[5] Abdelmoneim Amer Desouki, Florian Jarre, Gabriel Gelius-Dietrich, and Martin J Lercher. Cyclefreeflux: efficient removal of thermodynamically infeasible loops from flux distributions. *Bioinformatics*, 31(13):2159–2165, 2015.

[6] German Preciat, Agnieszka B Wegrzyn, Xi Luo, Ines Thiele, Thomas Hankemeier, and Ronan MT Fleming. Xomicstomodel: omics data integration and generation of thermodynamically consistent metabolic models. *Nature Protocols*, pages 1–42, 2025.

[7] Nicolás González-Arrué, Isidora Inostroza, Raúl Conejeros, and Marcelo Rivas-Astroza. Phenotype-specific estimation of metabolic fluxes using gene expression data. *Iscience*, 26(3), 2023.

2 Supplementary figures

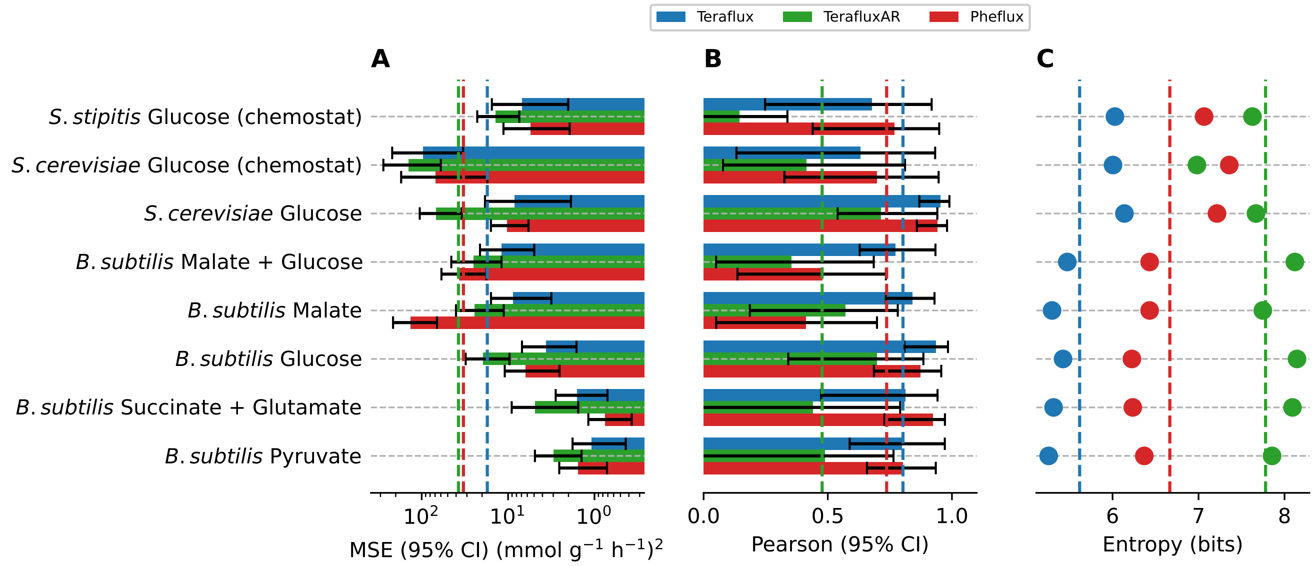

Figure S1: **Model performance and entropy analysis for different culture conditions.** Panel **(A)** shows the mean squared errors (MSE) between experimental and estimated metabolic fluxes by Teraflux (blue), the unconstrained control model TerafluxAR (green), and Pheflux (red), under different culture conditions. Likewise, panel **(B)** shows the Pearson correlation coefficients for these models. Panel **(C)** depicts the entropy values (in bits) calculated for the same metabolic fluxes. The dashed lines represent the average values across conditions for each model. The culture conditions evaluated vary in the limiting carbon source used. Confidence intervals (CI) were computed using a bootstrap procedure (n=1000).

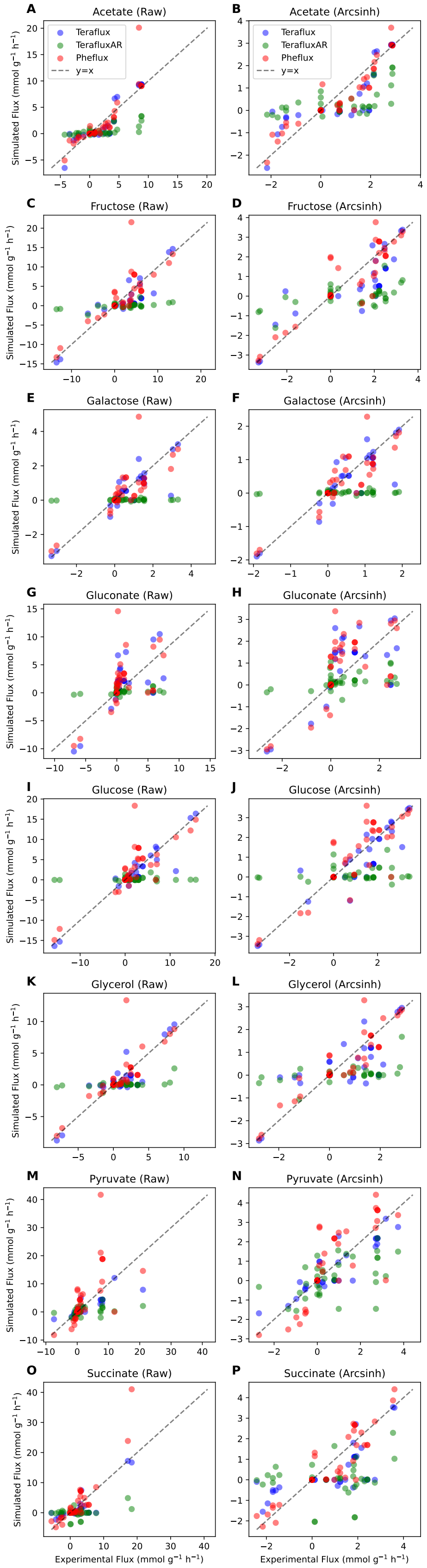

Figure S2: **Scatter plots for raw and arcsinh transformed data for *E. coli*** Panels **(A)** to **(P)** show the predictions of Teraflux and Pheflux for various culture conditions where different limiting carbon sources were used.

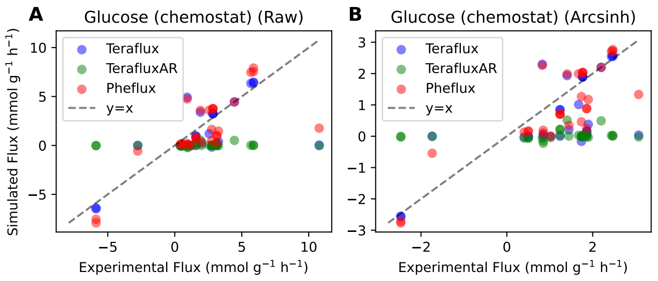

Figure S3: **Scatter plots for raw and arcsinh transformed data for *S. stipitis*** Panels **(A)** and **(B)** show the predictions of Teraflux and Pheflux for various culture conditions where different limiting carbon sources were used.

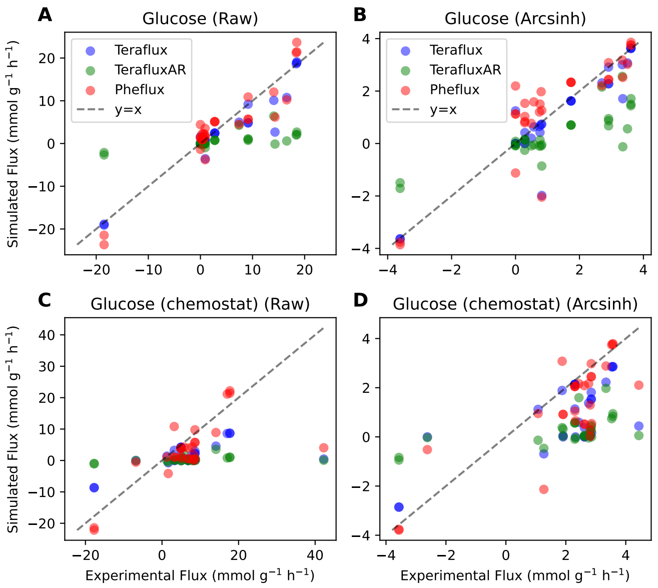

Figure S4: **Scatter plots for raw and arcsinh transformed data for *S. cerevisiae*** Panels **(A)** to **(D)** show the predictions of Teraflux and Pheflux for various culture conditions where different limiting carbon sources were used.

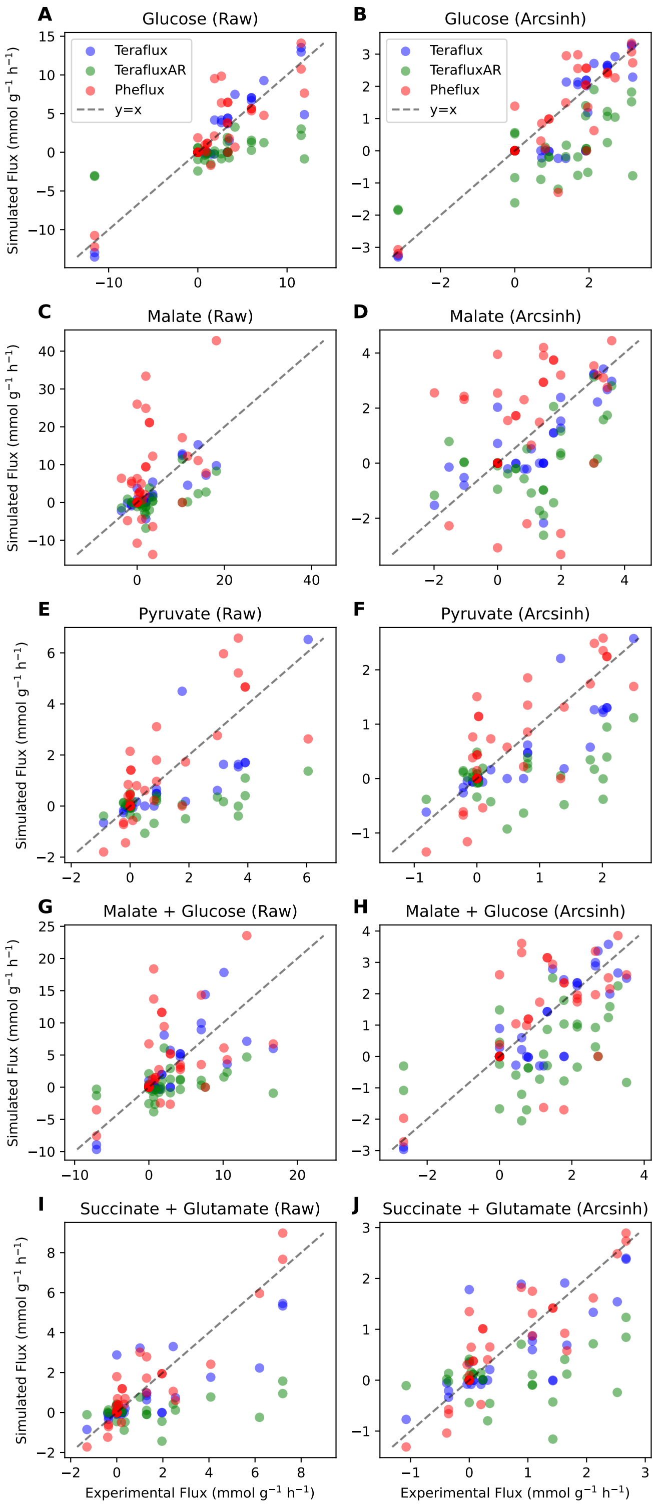

Figure S5: **Scatter plots for raw and arcsinh transformed data for *B. subtilis*** Panels **(A)** to **(J)** show the predictions of Teraflux and Pheflux for various culture conditions where different limiting carbon sources were used.

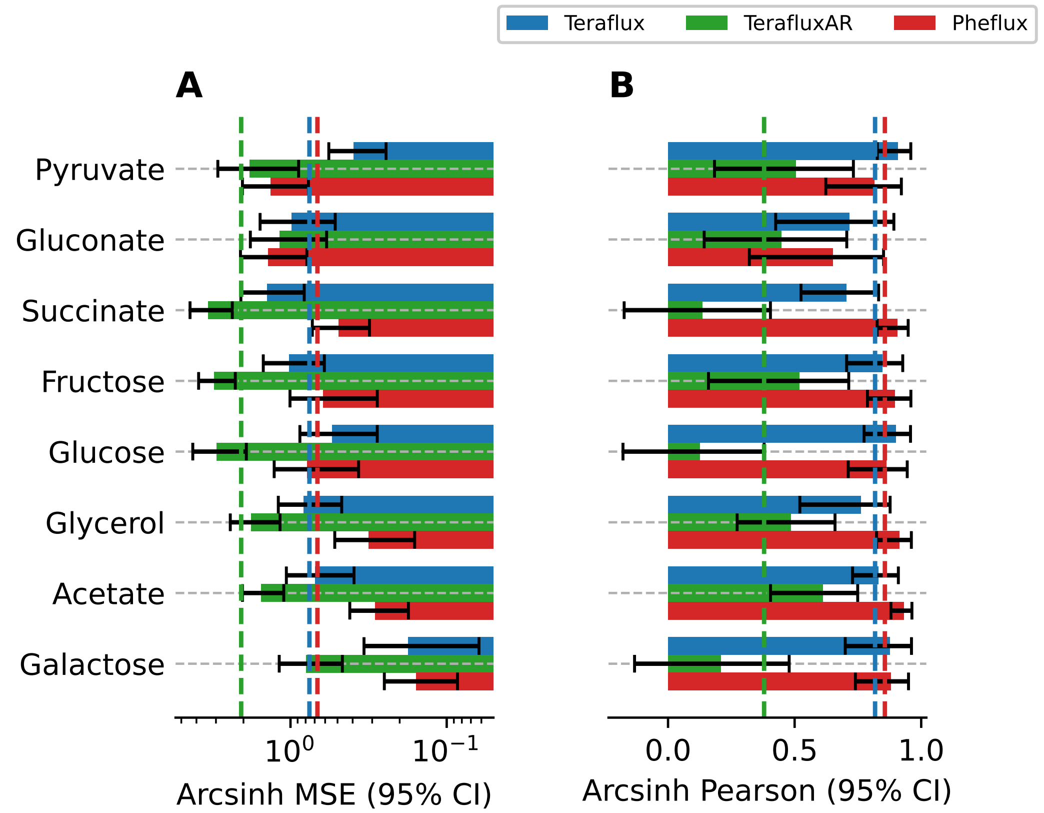

Figure S6: **Model performance for *E. coli* under different culture conditions on arcsinh-transformed data.** Panel **(A)** shows the mean squared errors (MSE) between experimental and estimated metabolic fluxes by Teraflux (blue), the unconstrained control model TerafluxAR (green), and Pheflux (red), under different culture conditions. Likewise, panel **(B)** shows the Pearson correlation coefficients for these models. The dashed lines represent the average values across conditions for each model. The culture conditions evaluated vary in the limiting carbon source used. Confidence intervals (CI) were computed using a bootstrap procedure (n=1000).

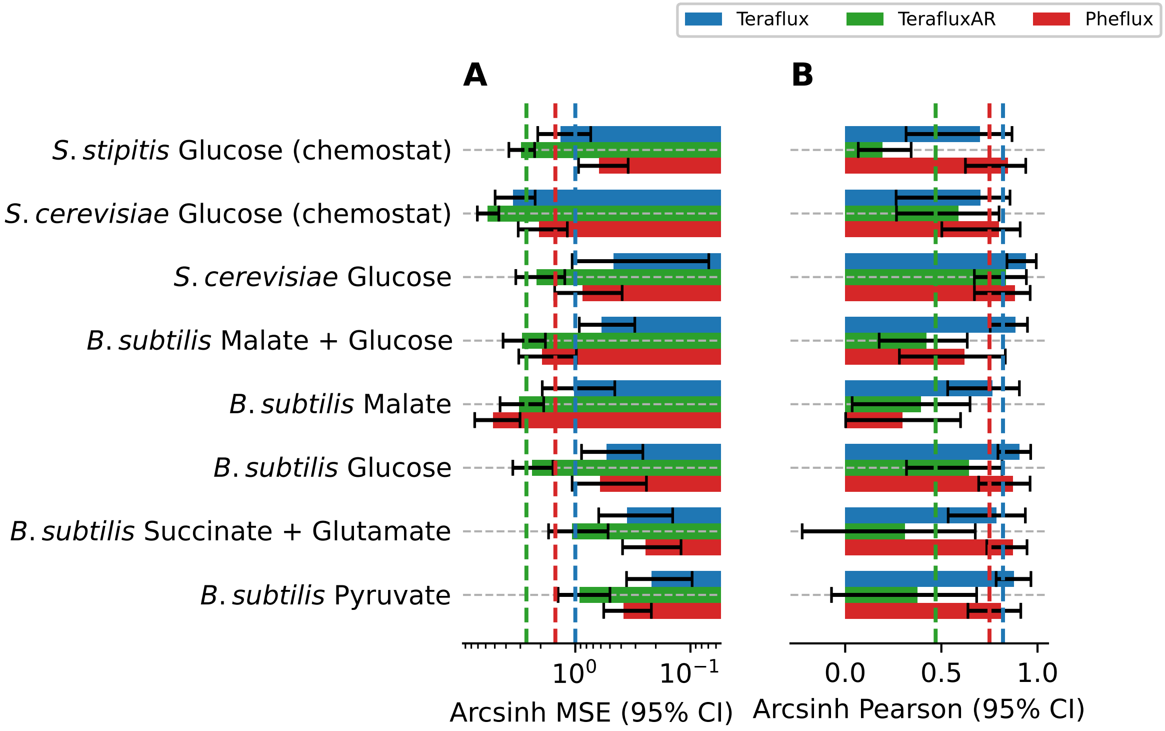

Figure S7: **Model performance for *S. stipitis* (1 condition), *S. cerevisiae* (2 conditions), and *B. subtilis* (5 conditions) on arcsinh-transformed data.** Panel **(A)** shows the mean squared errors (MSE) between experimental and estimated metabolic fluxes by Teraflux (blue), the unconstrained control model TerafluxAR (green), and Pheflux (red), under different culture conditions. Likewise, panel **(B)** shows the Pearson correlation coefficients for these models. The dashed lines represent the average values across conditions for each model. The culture conditions evaluated vary in the limiting carbon source used. Confidence intervals (CI) were computed using a bootstrap procedure (n=1000).
